# Antineuroinflammatory Activities and Neurotoxicological Assessment of Curcumin Loaded Solid Lipid Nanoparticles on LPS-Stimulated BV-2 Microglia Cell Models

**DOI:** 10.3390/molecules24061170

**Published:** 2019-03-25

**Authors:** Palanivel Ganesan, Byungwook Kim, Prakash Ramalaingam, Govindarajan Karthivashan, Vishnu Revuri, Shinyoung Park, Joon Soo Kim, Young Tag Ko, Dong-Kug Choi

**Affiliations:** 1 Department of Integrated Bioscience-Biomedical Chemistry, College of Biomedical and Health Science, Konkuk University, Chungju 27478, Korea; palanivel67@gmail.com; 2 Nanotechnology Research Center and Department of Integrated Bioscience-Biotechnology, Konkuk University, Chungju 27478, Korea; kbwxfile@gmail.com (B.K.); karthivashan@gmail.com (G.K.); ifresha@nate.com (S.P.); kgfdkr@gmail.com (J.S.K.); 3 Department of Medical & Molecular Genetics, Stark Neurosciences Research Institute, Indiana University School of Medicine, Indianapolis, IN 46202, USA; 4 Department of Pharmaceutical Sciences, Philadelphia College of Pharmacy, University of the Sciences, Philadelphia, PA 19104, USA; ramprabaprakash@gmail.com; 5 College of Pharmacy, Gachon University, Incheon 406-799, Korea; 6 Department of Green Bio engineering Korea National University of Transportation, Daehakro 750, Chungju 27469, Korea; vishnurevuri91@gmail.com

**Keywords:** lipopolysaccharide, solid lipid nanoparticle, curcumin, antineuroinflammation, toxicity, SEM

## Abstract

Curcumin, which is a potential antineuroinflammatory and neuroprotective compound, exhibits poor bioavailability in brain cells due to its difficulty in crossing the blood–brain barrier and its rapid metabolism during circulation, which decreases its efficacy in treating chronic neuroinflammatory diseases in the central nervous system. The bioavailability and potential of curcumin can be improved by using a nanodelivery system, which includes solid lipid nanoparticles. Curcumin-loaded solid lipid nanoparticles (SLCN) were efficiently developed to have a particle size of about 86 nm and do not exhibit any toxicity in the endothelial brain cells. Furthermore, the curcumin-loaded solid lipid nanoparticles (SLCN) were studied to assess their efficacy in BV-2 microglial cells against LPS-induced neuroinflammation. The SLCN showed a higher inhibition of nitric oxide (NO) production compared to conventional curcumin in a dose-dependent manner. Similarly, the mRNA and proinflammatory cytokine levels were also reduced in a dose-dependent manner when compared to those with free curcumin. Thus, SLCN could be a potential delivery system for curcumin to treat microglia-mediated neuroinflammation.

## 1. Introduction

Neurodegenerative diseases, such as Parkinson’s disease (PD) or Alzheimer’s disease, are age-related chronic illnesses that are characterized by the loss of neurons and activation of microglia in brain cells [1,2]. Numerous studies have confirmed that neuroinflammation is a major factor of the degeneration in nigral neurons that are important characteristics of neurodegenerative diseases [3]. Neuroinflammation leads to the activation of microglia and results in a higher production of inflammatory markers, including nitric oxide, TNF-α, IL-1β, and IL-6 [4,5,6,7]. The suppression of inflammatory markers using natural phytoextracts is in great demand for delaying neuroinflammation and its related diseases.

Phytoextracts have been used for generations as traditional treatments for neurodegenerative diseases [8,9,10,11]. Curcumin is a major bioactive compound that is found in turmeric powder and it is used in traditional Indian and Chinese medicine due to its various health enhancing effects to treat the inflammation associated with chronic diseases [12,13,14,15,16]. Furthermore, several in vitro and in vivo mechanisms have determined for the antineuroinflammatory pathway of curcumin. Its efficacy is still limited due to its low solubility, higher degradation by enzymes, lower absorption, and faster elimination [17,18,19,20,21]. To improve the properties and biological activity, novel lipid-based delivery vehicles are in greater demand [22], such as solid lipid nanoparticles (SLN). 

SLN delivery systems are highly efficient delivery systems for phytocompounds with less absorption and poorly bioavailability, such as curcumin, resveratrol etc. [23,24], due to their size and higher stability. Recently, some researchers studied the curcumin-loaded SLN and observed an enhancement in antitumor activity in vitro [25]. Furthermore, there was enhanced delivery of curcumin-loaded SLN to cells without altering the integrity of the cellular junction [26]. SLN is in greater demand compared to other nanodelivery systems due to its lower toxicity, ability to hold larger lipophilic compounds, and higher bioavailability and solubility [27,28,29]. SLNs were also reported to adhere to the endothelial cells of the blood–brain barrier (BBB) due to the surface adsorption of blood proteins, such as apolipoproteins and can effectively shuttle drugs across BBB. This was proved in a previous study, in which SLN was found to effectively shuttle the hydrophilic trypanocidal drug diminazene across the BBB. Our previous research confirmed that the curcumin-loaded SLN increased the bioavailability of curcumin in various organs, including the brain [30]. We further controlled the release of orally administered curcumin by developing a modified chitosan coated SLN that enhanced the bioavailability of curcumin [31,32,33,34]. To our knowledge, only a few studies have reported on the antineuroinflammatory role of curcumin-loaded SLN against lipopolysaccharide (LPS)-induced neuroinflammation in vitro and the molecular mechanism of its targeted pathways. Curcumin-loaded SLN is an important means to deliver curcumin to microglial cells in a highly bioactive way. Thus, the present study focused on the antineuroinflammatory mechanism of curcumin-loaded SLN (SLCN) and its possible pathway in LPS-induced microglial cells.

## 2. Results

### 2.1. Physical Properties of SLN and SLCN

The particle size of SLN and SLCN, PDI and zeta potential are shown in Table 1. The particle size of the SLCN was slightly higher compared to that of SLN, which means that the addition of curcumin slightly increased the particle size of the SLCN. However, the particle size was found to be lower than 100 nm and this could facilitate a higher bioavailability of curcumin to the cells. The EE and LC of SLCN were 98.8% and 3%, respectively. A very high lipophilic behaviour of curcumin (log P (Octanol/water partition coefficient) = 3.29) resulted in the higher intake of curcumin in the SLN solid lipid core, which is made up of only lipids. There were no traces of curcumin after centrifugation for EE and LC determination, with 98.8% of curcumin recovered from the SLN. The higher encapsulation might be due to the ability of SLN to strongly accommodate the lipophilic curcumin. We were able to confirm that curcumin has higher encapsulation efficiency in the SLN.

### 2.2. Scanning Electron Microscopy (SEM) of SLCN

**Figure 1:**
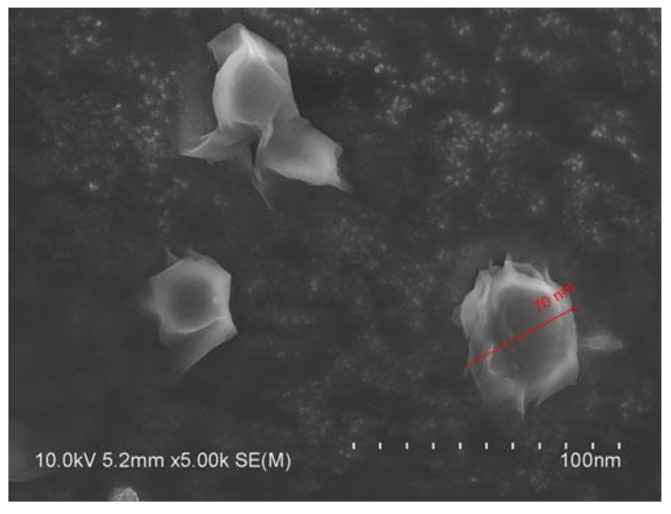
SEM micrograph of SLCN.

Furthermore, we confirmed the particle size of the dispersed SLCN in SEM and found that the SLCN has a uniform particle size of less than 100 nm with a spherical shape (Figure 1).

### 2.3. Cellular Toxicity Studies

**Figure 2:**
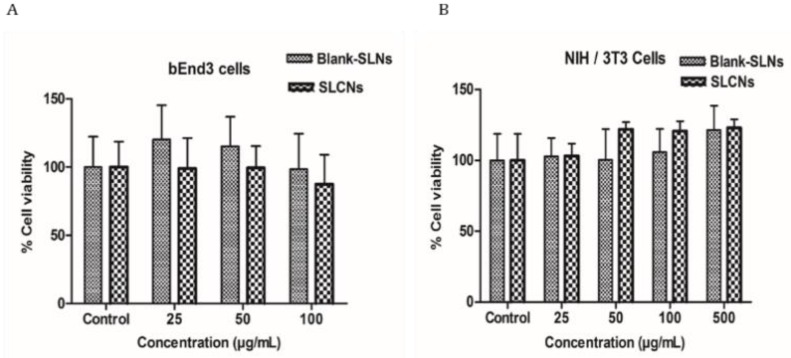
Cell viability of SLN and SLCN in bEnd3 cells (**a**)or NIH/3T3 cells (**b**).

The cell viability of the SLN and SCLN was compared between that in bEnd3 cells or NiH/3T3 cells, which is shown in Figure 2. The increase of about 100 μg/mL in the concentration of SLCN in brain endothelial cells showed no cellular toxicity while the increase of about 500 μg/mL in the NIH/3T3 cells showed no cellular toxicity for both SLN and SLCN. 

### 2.4. Effect of SLCN on NO Production in LPS-Stimulated BV2 Cells

**Figure 3:**
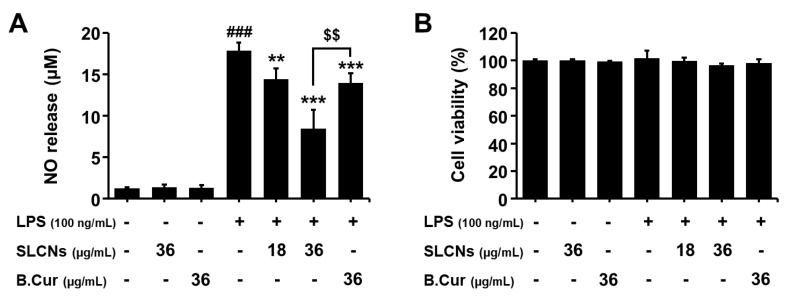
Effect of SLCN and base curcumin on NO production and cell viability in lipopolysaccharide LPS-stimulated BV2 cells. BV2 cells were pretreated with the indicated concentrations (18 and 36 µg/mL) of SLCN and base curcumin (B.Cur) for 1 h before incubation with LPS (100 ng/mL) for 24 h. Nitrite was measured using the Griess reaction (**A**). The cell viability was evaluated using the MTT assay (**B**). Results are displayed as a percentage of untreated groups. ###*p* < 0.001, vs. untreated group; **p < 0.01 and ***p < 0.001 vs. LPS-treated group; $$*p* < 0.01 vs. SLCN-treated group (one-way ANOVA; n = 4).

Most of the naturally-derived candidates were reported to be safe with negligible/null toxicity. Considering the potential of toxicity involved during formulation process in this study, we evaluated the cytotoxic effects of SLCN, base curcumin and/or LPS in BV2 cells. The cells were pretreated with SLCN (18 and 36 μg/mL) and base curcumin (36 μg/mL) for 1 h before the addition of LPS (100 ng/mL). The incubation with LPS alone markedly increased the NO production in the BV2 cells compared to the control (17.86 ± 0.98 µM; Figure 3A; ###*p* < 0.001 vs. untreated group). However, the pretreatment with SLCN and base curcumin prevented this increase in the levels of NO production in LPS-stimulated BV2 cells (Figure 3A; **p < 0.01 and ***p < 0.001 vs. LPS-treated group). Moreover, SLCN resulted in a significantly greater inhibition of NO compared to base curcumin for the same concentration (Figure 3A; $$*p* < 0.01 vs. SLCN-treated group). The cell viability was determined using an MTT assay. According to the results of our study, both drugs and LPS treated cells exhibited minimal/null toxic effects at the selected concentrations (Figure 3B).

### 2.5. SLCN Attenuated iNOS and COX -2 mRNA Expressions in LPS-Stimulated BV2 Cells

**Figure 4:**
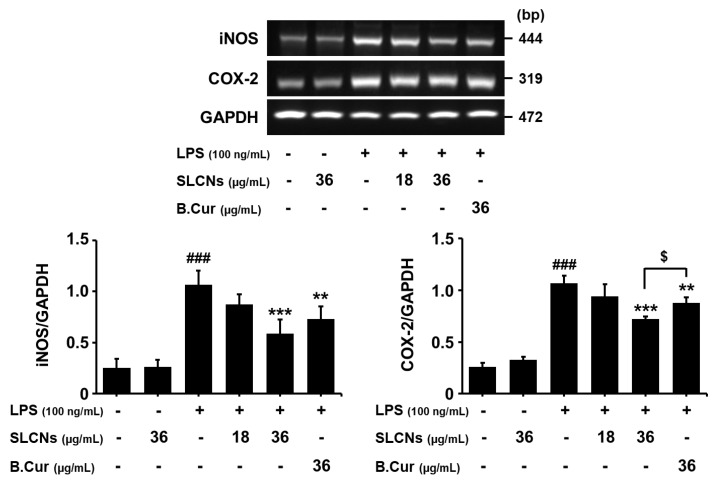
SLCN attenuates iNOS and COX-2 mRNA levels in LPS-stimulated BV2 cells. BV2 cells were pretreated with the indicated concentrations of SLCN and base curcumin for 1 h before being incubated with LPS (100 ng/mL) for 6 h (RT-PCR). Total RNA was prepared and analysed for iNOS and COX-2 gene expression by RT-PCR. Quantification data are shown in the lower panel. ###*p* < 0.001 vs. untreated group; **p < 0.01 and ***i < 0.001 vs. LPS-treated group; $*p* < 0.05 vs. SLCN-treated group (one-way ANOVA; n = 3).

BV2 cells were induced with LPS (100 ng/mL) in the presence or absence of SLCN and base curcumin at various concentrations (18 and 36 μg/mL, 36 μg/mL, respectively). LPS treatment significantly increased the iNOS and COX-2 mRNA levels (^###^*p* < 0.001 vs. untreated group) but pretreatment with SLCN and base curcumin at the indicated concentrations inhibited this LPS-induced mRNA expression of iNOS and COX-2 (Figure 4; ^**^p < 0.01 and ^***^p < 0.001 vs. LPS-treated group). Furthermore, SLCN improved the COX-2 mRNA level compared to the base curcumin at the same concentration (Figure 4; $*p* < 0.05 vs. SLCN-treated group). As discussed earlier, this can be attributed to the enhanced drug delivery potential of SLN. Thereby, as reported in earlier studies, the relatively enhanced bioavailability of curcumin released from SLCN is likely responsible for its higher suppression of iNOS and COX-2 mRNA expressions compared to its free form.

### 2.6. SLCN Inhibited the Production of Proinflammatory Cytokines in LPS-Induced BV2 Cells

**Figure 5:**
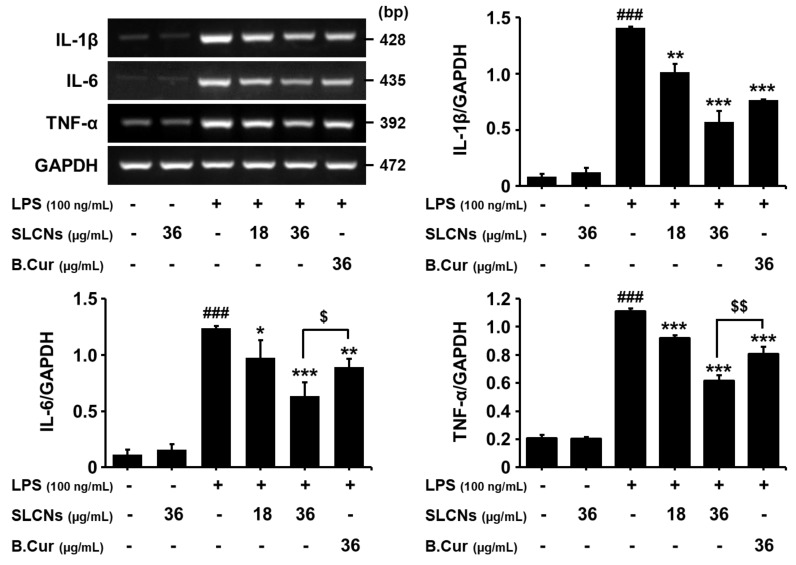
SLCN decreases the production of proinflammatory cytokines. Cells were pretreated with the indicated doses of SLCN and base curcumin for 1 h before LPS (100 ng/mL) treatment. The mRNA levels of TNF-α, IL-1β, IL-6 and GAPDH were determined via RT-PCR. There was a representative densitometry analysis of TNF-α, IL-1β and IL-6 compared with GAPDH mRNA, respectively. ###*E* < 0.001 vs. untreated group; *p < 0.05, **p < 0.01 and ***p < 0.001 vs. LPS-treated group; $p < 0.05 and $$*p* < 0.01 vs. SLCN-treated group (one-way ANOVA; n = 3).

RT-PCR results demonstrated elevated mRNA levels of these cytokines at 6 h after LPS treatment (^###^*p* < 0.001 vs. untreated group). Pretreatment with SLCN significantly inhibited the LPS-induced production of IL-1β, IL-6 and TNF-α (Figure 5; ^**^p<0.01 and ^***^p<0.001 vs. LPS group). Furthermore, SLCN resulted in a greater reduction in the IL-6 and TNF-α mRNA levels compared to the base curcumin at the same concentration (Figure 5; ^$^*p* < 0.05 and ^$$^*p* < 0.01 vs. SLCN-treated group). Thus, our results indicate that SLCN inhibited the expression of proinflammatory mediators and associated cytokines involved in the inflammatory process. 

## 3. Discussion

The research aim of this study was to enhance the delivery of curcumin to the brain cells and to study the possible antineuroinflammatory molecular mechanisms and neurotoxicological effects of the curcumin loaded in SLN. SLCN created with different lipids had higher particle sizes [30], which confirmed that the lipids in SLN play a significant role in determining the particle size of the SLCN. The incorporation of curcumin also causes a slight increase in the PDI and the zeta potential of the SLCN, with similar results observed in other studies [19,30]. The higher encapsulation might be due to the ability of SLN to strongly accommodate the lipophilic curcumin. The results were consistent with our previous studies [32]. Furthermore, the toxicity evaluation showed that a higher concentration of curcumin loaded in SLN resulted in lower toxicity towards the brain endothelial cells. Similar results were also shown in other studies with a higher concentration of SLCN, which showed no cellular toxicity [34].

Furthermore, we studied the antineuroinflammatory mechanism of curcumin-loaded SLN in LPS-induced BV2 microglial cells. The macrophage-like microglia are the resident innate immune cells of central nervous system, which form the first-line defense to the CNS from the invading candidates via several inflammatory signaling pathways [35]. The activation of microglial cells, either acute or chronic, release several proinflammatory signals and cytotoxic factors, such as nitric oxide, cytokines and ROS, to efficiently eradicate the invaded neuronal cells and safeguard the spread of disease conditions. Despite this, the glial cells were also reported to facilitate neuroprotective properties to regulate and resolve the inflammatory conditions by promoting several anti-inflammatory mediators and cytoactive growth factors to repair the cells. Thus, it plays a dual role in both the destruction and protection of neuronal cells. The homeostasis of the neuroinflammation is balanced by a feedback loop, which limits the collateral damage inflicted by the inflammatory response. However, the imbalance of this inflammatory homeostasis leads to an overproduction of NO, which subsequently cascades several neuroinflammatory disorders. This is consistent with previous studies where the nanolipid drug formulations exhibit better NO inhibitory activity in LPS-stimulated macrophages compared to its free form. This is likely due to the enhanced drug delivery potential. Although most of the naturally-derived candidates were reported to be safe with negligible/null toxicity, considering the potential toxicity involved during the formulation process in this study, we evaluated the cytotoxic effects of SLCN, base curcumin and/or LPS in BV2 cells. The cell viability was determined using an MTT assay. According to the results of our study, both drugs and LPS treated cells exhibited minimal/null toxic effects at the selected concentrations.

**Figure 6:**
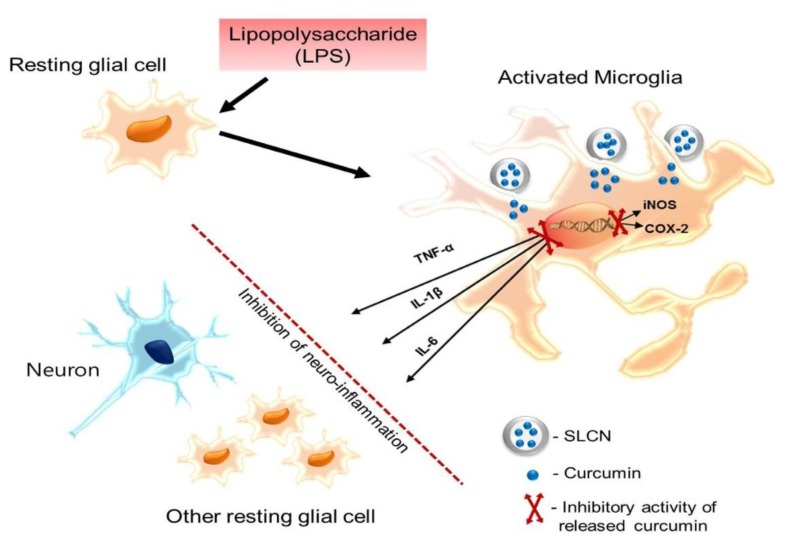
Postulated SLCN antineuroinflammatory mechanism of action in LPS-stimulated BV2 cells.

At the inflammation site, the activated microglial cells were reported to elevate the proinflammatory mediators that are commonly generated by the inducible isoforms of NO synthase (iNOS) and cyclooxygenase-2 (COX-2) enzymes. Several scientific evidences reported that the inducible forms of iNOS and COX-2 facilitates the inflammatory conditions by the production of surplus NO and prostaglandins (PG), respectively, which are highly cytotoxic. Interestingly, the induction of both iNOS and COX 2 were bound to the transcriptional regulation. In our study, the relatively enhanced bioavailability of curcumin released from SLCN is likely responsible for its higher suppression of iNOS and COX-2 mRNA expression compared to its free from. The activated microglial cell releases proinflammatory mediators (iNOS and COX 2) and further facilitates the transcriptional cascades to elevate the supply of downstream proinflammatory cytokines, such as IL-1β, IL-6 and TNF-α [36]. These released proinflammatory cytokines were reported to synergistically exacerbate the inflammatory milieu together with NO and PGs by activating the neighboring glial cells and astrocytes. Thus, they play pivotal roles in microglia-mediated inflammation. It was previously reported that LPS induction substantially elevates the upsurge of proinflammatory cytokines (IL-1β, IL-6 and TNF-α) via the MAPK signaling pathway in BV-2 glial cells. Relatively, the candidates that suppress the expression of these proinflammatory cytokines were reported to show substantial neuroprotective effects against several inflammatory induced neurodegenerative disorders [37]. This is consistent with previous study findings where the active drug candidates loaded into nanoparticles resulted in substantial suppression of proinflammatory cytokines and exhibited relatively higher antineuroinflammatory effects than their free forms. Another recent study showed that curcumin exerts an antiinflammatory effect by downregulating the PI3k/Akt signaling pathway and NF-κB (nuclear factor kappa-light-chain-enhancer of activated B cells) protein levels [38]. This could possibly be attributed to the downregulation of mRNA expression of proinflammatory cytokines by SLCN in our current study. It is also noteworthy that a previous study report states that SLN itself does not impact the viability and inflammatory effects of macrophages [39]. Thus, our results indicate that curcumin-loaded SLCN inhibited the expression of proinflammatory mediators and associated cytokines involved in the inflammatory process. The possible antiinflammatory mechanism of the SLCN in LPS that stimulated BV-2 microglial cells is depicted in Figure 6.

## 4. Materials and Methods 

### 4.1. Materials

Curcumin (*C. longa. Linn*) was obtained from TCI Co., Ltd. (Tokyo, Japan), Lipopolysaccharide (LPS, E. coli 0111:B4), dimethyl sulfoxide (DMSO), sulfanilamide, N-(1-naphthyl)-ethylenediamine dihydrochloride 3-(4,5-dimethylthiazol-2-yl)-2,5-diphenyltetrazolium bromide (MTT) and Tween-20 were purchased from Sigma–Aldrich (St. Louis, MO, USA). Foetal bovine serum (FBS), Dulbecco’s Modified Eagle Medium (DMEM), penicillin-streptomycin (P-S), trypsin/EDTA (TE), TRIzol and other cell culture plates were obtained from Gibco-BRL (Rockville, MD, USA).

### 4.2. Solid Lipid Curcumin Nanoparticle Formulation and Optimization

The formulation of curcumin-loaded SLCN was prepared using high shear homogenization and ultrasonication techniques, with the different lipid formulations of SLCN used as shown in Table 1. This was conducted according to the modified method of Ramalingam and Ko Briefly, different ratios of precirol, palmitic acid and gelucire were heated to 85 °C to complete solubilize the lipid. After the lipid was completely melted, curcumin was added to the lipid phase. The aqueous phase containing Tween 80 was added to the lipid phase and homogenized using Ultra- Turrax homogenizer (IKA-Werke, Staufen, Germany) at around 11,000 rpm for 5 min. The curcumin or free preemulsion was further ultrasonicated for 5 min using a probe sonicator (Vibracell VCX130; Sonics, Newtown, CT, USA). The obtained SLCN was cooled for 30 min and lyophilized before being stored at 4 °C for further experiments.

### 4.3. Particle Size, Zeta Potential and Polydispersity Index 

The particle size, zeta potential and polydispersity index (PDI) of SLN and SLCN was measured using an ELSZ-1000 zeta potential and particle size analyser (Photal OTSUKA Electronics, Tokyo, Japan). The SLCN efficiency of EE and LC was measured according to the modified method as described by Ramalingam and Ko [30]. For SEM, 10 µL of the 0.1 mg/mL SLN sample was added to the silica grid, dried, sputter coated with platinum and further used for HR-SEM analysis (High Resolution Field Emission Scanning Electron Microscope JSM-7610F, JEOL Ltd., Akishima, Tokyo, Japan). 

### 4.4. Cell Toxicity Studies

Cellular toxicity studies of SLCN and SLN were carried out and compared using two different cells, such as NIH/3T3 and brain endothelial cells, using an MTT assay. 

### 4.5. Cell Culture, NO Release Assay and Cell Viability

BV2 cells were cultured in DMEM supplemented with 5% FBS and 100 U/mL P-S before being maintained in a humidified incubator in an 5% CO_2_ atmosphere. In all experiments, cells were seeded at a density of 5 × 10^5^ cells/mL and were pretreated for 1 h with the indicated concentrations of curcumin-loaded solid lipid nanoparticles (SLCN) and base curcumin before being incubated in a medium containing LPS (100 ng/mL). The LPS-induced release of NO in the culture medium was determined and the cell viability of the cultured cells was measured. Briefly, the seeded cells were treated with different concentrations of SLCN (18 and 36 µg/mL) and 36 µg/mL of base curcumin, which was followed by incubation with LPS for 24 h. After incubation with LPS for 24 h, 50 μL of each culture medium was mixed with 50 μL of Griess reagent. Nitrite levels were determined using a microplate reader at 540 nm (Tecan Trading AG, Switzerland) and nitrite concentrations were calculated by reference to a standard curve generated by the known concentrations of sodium nitrite. The cell viability was measured by adding 0.5 mg/mL of MTT to each well. After incubation for another 4 h at 37 °C and 5% CO2, the medium was removed from each well and the formazan crystals that formed were dissolved in DMSO. The absorbance was determined at 540 nm using a microplate reader (Tecan Trading AG).

### 4.6. Reverse Transcription-Polymerase Chain Reaction (RT-PCR) Viability

BV2 microglia cells were plated overnight in 6-well culture plates and were pretreated for 1 h with the indicated concentrations of SLCN (18 and 36 µg/mL) and base curcumin (36 µg/mL) before incubation in a medium containing LPS (100 ng/mL). The total RNA was extracted using TRIZOL (Invitrogen). RNA (1 µg) was reverse-transcribed using ReverTra Ace-α kit (Toyobo, Osaka, Japan) according to the manufacturer’s instructions. The inducible nitric oxide synthase, cyclooxygenase type 2 (COX-2), tumour necrosis factor-alpha (TNF-α), interleukin 1β (IL-1β), IL-6 and glyceraldehyde 3-phosphate dehydrogenase (GAPDH) genes were amplified from the cDNA via polymerase chain reaction (PCR). cDNA was amplified by PCR using the specific primers mentioned in Table 2. PCR was performed using an initial step of denaturation (5 min at 94 °C), 20–27 cycles of amplification (94 °C for 30 s, 54–58 °C for 1 min and 72 °C for 1 min) and an extension (72 °C for 5 min). PCR products were analysed on 1.5% agarose gels with EtBr stained. The mRNA of GAPDH served as an internal control for sample loading and mRNA integrity. The band intensity was quantified via a densitometry analysis using multi-gauge software V3.1 (Fujifilm, Tokyo, Japan).

### 4.7. Statistical Analysis

The values given are means ± S.E.M. of at least three separate experiments conducted in triplicate. The comparisons between groups were analyzed using a one-way analysis of variance (ANOVA) followed by Tukey’s multiple comparison test using software GraphPad Prism V6.01 (GraphPad Software Inc., San Diego, CA, USA)

## 5. Conclusions

The antineuroinflammatory potential of curcumin-loaded solid lipid nanoparticles was studied in this work. SLCN effectively and dose-dependently reduced the NO release in LPS-induced BV-2 microglial cells, which potentially occurred via the regulation of proinflammatory cytokines and associated mediators. Interestingly, the curcumin released from SLN exhibited a relatively higher inflammation-suppressive activity compared to its free form, which can be likely attributed towards the sustained release and enhanced bioavailability of SLN. Thus, further studies on fabrication and functionalization of SLCN can effectively pave the way to develop novel drug candidates to treat inflammation-mediated neurodegenerative disorders.

## Figures and Tables

**Table 1:** Physical properties of solid lipid nanoparticles (SLN) and curcumin-loaded solid lipid nanoparticles (SLCN).

Formulations	Particle size (nm)	PDI	Zeta Potential (mV)	EE (%)	LC (%)
SLN	83.16 ± 1.24	0.27 ± 0.02	−24.29 ± 1.66	-	-
SLCN	86.60 ± 9.85	0.29 ± 0.02	−22.15 ± 1.32	98.8 ± 1.00	3.01

SLN: solid lipid nanoparticle; SLN: Curcumin loaded solid lipid nanoparticle; PDI: poly dispersity index; EE: Encapsulation efficiency; and LC: Loading capacity.

**Table 2:** Primer sets used for RT-PCR.

Gene	Primer Sequence		Size (bp)	Accession
**iNOS**	Forward	5′-CTTGCAAGTCCAAGTCTTGC-3′	369	NM_010927
	Reverse	5′-GTATGTGTCTGCAGATGTGCTG-3′		
COX-2	Forward	5′-ACATCCCTGAGAACCTGCAGT-3′	414	NM_011198
	Reverse	5′-CCAGGAGGATGGAGTTGTTGT-3′		
IL-1β	Forward	5′-CATATGAGCTGAAAGCTCTCCA-3′	385	NM_008361
	Reverse	5′-GACACAGATTCCATGGTGAAGTC-3′		
IL-6	Forward	5′-GGAGGCTTAATTACACATGTT-3′	435	NM_031168
	Reverse	5’-TGATTTCAAAGATGAATTGGAT-3‘		
TNF-α	Forward	5′-TTCGAGTGACAAGCCTGTAGC-3′	390	NM_013693
	Reverse	5′-AGATTGACCTCAGCGCTGAGT-3′		
GAPDH	Forward	5′-CCAGTATGACTCCACTCACG-3′	378	GU214026
	Reverse	5′-CCTTCCACAATGCCAAAGTT-3′		
